# The need for a new perspective on decision-making in bacteria

**DOI:** 10.1080/19420889.2025.2463926

**Published:** 2025-02-12

**Authors:** Sibin Mathew Nesin, Mathew Chandrankunnel

**Affiliations:** a Department of Psychology, School of Psychological Sciences, CHRIST University, Bangalore, India; b Dharmaram Vidya Kshetram, Bangalore, India

**Keywords:** Bacterial consciousness, bacterial decision- making, cognitive flexibility, consciousness, individual behavior, microbial cognition, microbial intelligence, the cellular basis of consciousness (CBC)

## Abstract

The individualistic and collectivistic intelligent behaviors observed in mammals, birds, and fishes have been appreciated by many scientists in recent years and supported by the Cambridge Declaration on Consciousness in 2012. Behavioral studies in lower organisms like arthropods and cephalopods showed the presence of multisensory integration, decision-making, and goal-directed behavior in these non-vertebrate animals. The presence of intelligent and history-dependent behaviors has been studied in microorganisms, and recent studies propose the possibility of cognition in single cellular organisms. The Cellular Basis of Consciousness (CBC), proposed by Arthur Reber in 2016 and elaborated by Baluška and Reber in 2019, suggests the possibility of consciousness in single cellular organisms. However, the critics of the Cellular Basis of Consciousness theory state that the individual bacterial cell does not make choices, and the decision-making is the result of stochastic differences in protein levels. Here, we want to address the criticism of decision-making in bacteria. An attempt is made to give a new perspective to the existing model to explain the flexibility in bacterial behavior in an ever-changing environment. The authors would like to consider an alternative perspective on flexibility in decision-making as the result of multiple pathways that have convergence and divergence as observed in the brain. Flexibility provides the possibility to have individualistic behavior, and the existence of such pathways can be considered as the molecular mechanism underlying individualistic decision-making in bacteria as well as in humans.

## Introduction

The individualistic and collectivistic intelligent behaviors observed in mammals, birds, and fishes have been appreciated by many scientists in recent years [1] and supported by the Cambridge Declaration on Consciousness in 2012. Behavioral studies in lower organisms like arthropods and cephalopods showed the presence of multisensory integration, decision-making, and goal-directed behavior in these non-vertebrate animals. The natural emergence of consciousness [2] proposes that phenomenal consciousness emerged during the Cambrian explosion period (about 520 to 560 million years ago), resulting from multilevel sensory processing, integration, and information storage. The presence of intelligent and history-dependent behaviors has been studied in microorganisms, and recent studies propose the possibility of cognition in single-cellular organisms [3]. The Cellular Basis of Consciousness (CBC), proposed by Arthur Reber in 2016 and elaborated by Baluška and Reber in 2019 [4], suggests the possibility of consciousness in the single cellular organisms.

The CBC theory states that “all living organisms are conscious, self-aware, and have valenced sensory, and perceptual experiences” [5]. The critics of the CBC point out that the individual cells behaving differently can be traced to stochastic differences in protein levels between cells and the bacteria responding to the environment through the signaling pathways that should not be considered as conscious decision-making but mere response, which is highly specific. The critics of the CBC emphasize that consciousness is absent in the lower organisms, especially single cellular organisms, and that the development of the nervous system might have led to the emergence of consciousness [6].

Here, we want to address the criticism of decision-making in bacteria which is based on individualistic and flexible approaches. An attempt is made to give a new perspective to the existing model to explain the flexibility of individual bacteria’s behavior in responding to environmental changes. Various examples are given to explain the presence of individualistic and collectivistic decision-making in bacteria and the molecular mechanism underlying such behaviors. The presence of converging and diverging pathways is observed in bacteria and such converging and diverging molecular as well as neuronal pathways are present in the brain. The cases of skilled movement and cognitive flexibility can be studied to understand the presence of converging and diverging pathways (both neural pathways and cell signaling pathways) in the human brain. The new perspective on decision-making in bacteria and humans as the result of converging and diverging pathways that provide individual flexibility points to the presence of conscious decision-making in bacteria.

## Molecular mechanisms underlying individualistic and colony behavior in bacteria

The neural network’s molecular mechanism is much more complex than the molecular mechanism underlying the bacterial behavior. Many recent research studies have addressed the molecular mechanism underlying bacterial behavior [3]. There are about 10,000 chemoreceptors per cell of *E. coli*, and each of these chemoreceptors has multiple binding sites [3]. To survive in an environment, bacteria have to monitor the physicochemical conditions in their environment constantly. The ability of bacteria to monitor physicochemical changes in the environment is the result of the response of various receptors. Receptors like bacterial phytochrome receptors (BphP) detect the presence of light; pH variation, detected by electrochemical properties and serine receptor, Tsr; the temperature variation, by Tar and Tsr receptors; presence of sugars by Trg; and presence of oxygen by FixL receptors are examples of such receptors in bacteria. Stimulus elicits a specific pathway through signal transduction that results in the changes in gene expression or activation of specific pathways that ultimately lead to the change in behavior. Studies on the chemotaxis of *E. coli* have shown the individualistic variation in flagellar movement despite the genetic similarity of the clonal population. Even though the behavior observed is simple, the underlying molecular mechanism is complex, as in the case of chemotaxis in bacteria [7] or the case of skilled movement in mammals [8].

The receptors interact and elucidate a response with the help of signaling pathways, one-component and two-component systems, in bacteria. One component system like LuxI- LuxR has been found to be present in various bacterial strains. Thousands of LuxR-type receptors have been identified. Many bacterial species have different types of LuxR-type receptors, and one bacterium may have many kinds of LuxR receptors. The gene to produce the corresponding inducer (LuxI) for the many LuxR-type receptors is usually absent in the bacterium, and these receptors are called LuxR-solos. A bioinformatic survey revealed that many proteobacterial and non-proteobacterial genomes carry QS domain LuxRs, indicating the presence of multiple LuxR solos and indicating that evolution had incorporated the genes for the expression of various LuxR-type which could recognize the LuxI produced by other species of bacteria. Some LuxR-type receptors have species specificity and interact only with inducers from specific bacterial species. The two-component signaling also influences community behavior. The sensor histidine kinase receptor LuxPQ mediates the individualistic behaviors in bacteria in an unliganded state. In low cell density (LCD), the LuxQ acts as a kinase and at high cell density (HCD), it acts as a phosphatase. The HCD results in the accumulation of autoinducer-2 (AI-2), an inducer of LuxPQ. When the AI-2 binds to the LuxPQ, it inhibits the individualistic behavior and thus leads to the colony behavior. A quorum-sensing two-component receptor LuxN shows specificity in the ligand binding. The interspecies interaction can also happen through the two-component signaling pathway, especially between a host and the invading bacteria. For example, the disruption of tight junctions in mammalian epithelial cells can lead to the secretion of a mimic of the AI-2 molecule that can act on the bacterial receptor. It was shown that the mimic of the AI-2 molecule produced by the epithelial cells binds at the same site where AI-2 binds to the LuxP receptor. Similar eavesdropping of events happening in the host system was also found in other cases. Quorum sensing is a complex system of bacterial interaction, and the interaction of signaling molecules provides flexibility and adaptability of bacterial individualistic and colony behavior. A detailed report on such elaborate interaction can be found elsewhere [9,10] and outside the scope of the present article.

## Bacterial interaction with the environment

In unfavorable conditions, the bacteria and other microorganisms enter into a stationary phase. Most bacteria in the natural environment remain in the stationary phase or G0 phase. The cell wall becomes thicker with the increase in the peptidoglycan layer, ribosomes become 100S and inactive, and nucleoid condensation protects the DNA and reduces the cell metabolism. In the stationary phase, various bacteria have different fates. Some may remain in the stress resistance phase; some remain as biofilm, which is not multiplying; and some form endospores. If the stationary phase continues, bacteria may enter into a viable but non-culturable state (VBNC), growth advantage in the stationary phase (GASP), constant activity stationary phase (CASP), or stationary phase contact-dependent inhibition (SCDI) phase. Activation of each phase depends on the species of bacteria and the environmental conditions. For example, in the *Escherichia coli* SCDI phase, physical contact between the mutant and the wild-type bacteria is necessary. Stress factors like low nutrients cause gene mutations in the glycogen synthesis pathway. These mutants produce excess amounts of glycogen to survive the harsh environmental conditions and also kill the wild-type bacteria from which they originated. These mutations are driven by the changes in the environment and do not happen by chance alone. The mutation of a single gene is aimed at producing a specific characteristic (glycogen synthesis) and that mutation has altered the behavior of the bacteria to express cannibalism (because for glycogen synthesis, the bacteria need nutrients, and the only nutrient available in the low nutrient condition is the wild-type bacteria).

Apart from the behaviors observed in bacteria, studies have shown similarity of structure and function of various proteins with humans. The G-protein coupled receptors (GPCRs) in eukaryotes and microbial sodium-translocating rhodopsins have strikingly similar three-dimensional structures. Various ion channels such as mechanosensitive channels, K^+^ channels, Na^+^ channels, and Cl^−^ channels have evolutionary origin from bacteria and archaebacteria. The structural and functional similarity indicate the evolutionary origins of ion transport in the cell. For example, a family of prokaryotic inward-rectifier K+ channels (KirBac’s) have sequence similarity to eukaryotic homologue. The 12-pS channel in *Bacillus halodurans* is similar to L-type Ca^2+^ channel in animals and can be inhibited by the same blockers. However, in bacteria, the 12-pS channel is selective for Na^+^, not Ca2^+^, and is named NaChBac. The ligand-binding domain in metabotropic glutamate receptors is related to bacterial periplasmic-binding protein. Proteomic studies have shown that pathogenic bacteria, as compared to nonpathogenic bacteria, have more similarity in the proteome to human proteome than previously thought. The recent discoveries in the gut-brain axis show bidirectional communication between the microbes in gastrointestinal tract and the central nervous system.

Long-term anticipatory behaviors are observed in bacteria. *E. coli* survives in the mammalian gut, which is warm and anaerobic. Studies have found that the increase in temperature also switches on the genes that are essential for the bacteria to survive in anaerobic conditions even though the present state is aerobic [11]. Long-term memory stored by generations prepared the bacteria to produce anticipatory behaviors. Such anticipatory behaviors are ubiquitous in the microbial world. Another example is *Vibrio cholera*, which prepares to live outside the host in the last stage of infection. The bacteria can learn to switch between different sugars depending on the gut condition. A theoretical model was designed to demonstrate the dynamic mechanisms that might help the bacteria to survive unpredictable environments [12]. The phenotypic heterogenicity depends on stochastic switching across different states, helping the bacteria withstand unpredictable environmental fluctuations. The stochastic switching of gene expression requires cellular memory as a crucial component. Cellular memory produces more accurate and beneficial decision-making. The swarming behavior in *E. coli* was found to be affected by previous exposure, and the memory of this behavior can be passed on to four generations. Iron-based signal transduction was found to have a crucial role in cellular memory and decision-making, and such iron memory can integrate multiple stimuli and thus can affect multiple pathways.

## Multiple pathways in bacteria and their interconnectedness

Signaling in bacteria is not an independent event but an interaction of interconnected pathways similar to mammalian signal transduction. This interconnection results in regulatory control of signaling molecules and coincidence detection. For example, the binding of AI-2 and CAI-1 (cholera autoinducer 1) to the LuxPQ receptor and CqsS receptor, respectively, will only activate the HCD behaviors. If one of the receptors is in the inactivated state, it will not inhibit the individualistic behaviors or activate the colony behavior. Thus, the receptors LuxPQ and CqsS simultaneously by AI-2 and CAI-1 will lead to the activation of colony behavior in *Vibrio cholera* biofilm.

The studies on various bacteria revealed that quorum sensing promotes the cells to behave in a collective manner. The feedback mechanism of the pathways involved in HCD behavior functions in a coordinated manner to produce the desired outcome. Apart from the phosphate regulated receptor signaling cascades, the bacterial cell has another regulatory mechanism of gene expression like the sigma (σ) factors. The σ factors are regulators of RNA Polymerase (RNAP) function. These transcriptional regulatory proteins are subunits of RNAP, and they regulate the transcription via transcription initiation, recognition and opening of promoter region in the gene. To regulate the concentration and association of σ factors, there are anti-σ-proteins. These anti-σ-proteins either control the synthesis of σ factors or cause proteolysis of σ factors. Each strain of bacteria has different types of σ-factors and anti-σ-factors, increasing the flexibility in behavior. The concentration of these regulatory factors is controlled by the molecules inside and outside the cell. In *Bacillus subtilis*, σB -factor causes the activation of more than 150 genes in response to various environmental stress. In normal conditions, the concentration of σB -factor regulates the gene expression via sustained pulsing. Instead of keeping a stable concentration over time, the stochastic pulsing of the concentration of σB-factor has a specific frequency of higher and lower concentration. Studies have shown that the stress factors cause the bacterial cell to undergo modulation of the frequency of these pulses. The cellular concentration of σ -factors and anti-σ-factors thus regulate the gene expression and thus the cell functioning in relation to external and internal stimuli. Extracytoplasmic function σ-factors (ECFs) play a crucial role in stimulus perception and expression of corresponding genes, and studies have shown the complex relationship in signaling between the bacteria and the environment.

The regulatory networks of gene expression in bacteria are interconnected. For example, the arginine biosynthetic pathway is dispersed over the chromosome, which is controlled by the same regulatory protein, the arginine biosynthesis repressor (ArgR). Most of the cellular processes in bacteria require the coordinated expression of tens of genes, which are scattered at different locations in the bacterial chromosome and can be regulated by a same protein called regulon [13]. The regulatory proteins which control genetic expression do not follow a one-to-one path but have converging and diverging nodes [13]. The systemic understanding of the regulatory network consists of complex circuits of these regulatory interactions. The interaction of these networks is not random but follows modular principles. A module is a group of genes functioning in a coordinated manner to facilitate a function. Such complex system-level interaction and its regulation, which depends on internal and external environmental cues, are still under exploration. The effector level of such pathways can vary according to the input signal (cue). Our understanding of the regulation of gene expression in bacteria grew from operon to regulon to module.

The factors in bacteria that cause individual variations in response to changes in a natural environment are still under exploration. These complex interactions of modules with converging and diverging nodes provide multiple pathways in molecular signaling, hence flexibility in behavior and individual variation. In the article, we have ignored the individual variation arising from the variation in genes present in the bacterial chromosome and plasmids. Presence of multiple pathways with converging and diverging nodes is present in the human nervous system. The multiple pathways observed in the human brain are the result of intriguing neural networks. The molecular mechanism of such neural network is the presence of electrical and chemical synapses that activate a cascade of molecular pathways in neurons. The molecular mechanism underlying memory is well studied and the presence of converging and diverging molecular and neural pathways leads to the formation of different forms of implicit and explicit memory.

## Multiple pathways provide flexibility

Studies on skilled movement have provided evidence for the existence of more than one pathway that helps us to prepare multiple competing movements in an everchanging environment [14]. The unpredictability of the action required to achieve the goal prepares the brain to develop multiple pathways that can be used in a flexible manner. The development of such pathways occurs during motor learning, and the flexibility increases with the experience of attaining competing options [14]. The labile motor plans in the brain provide flexibility, efficiency and better accuracy in motor performance, and these multiple pathways can interact with each other [8]. The converging nodes and divergence in the neural network facilitate the transient shift from one pathway to another. The cognitive and behavioral flexibility of the neural process helps in adaptive flexibility and individualistic outcomes. The existence of multiple pathways facilitates the integration and provides alternative troubleshooting. The pathways can be converging and diverging and it provides possibility to have varying behavioral outcomes. The major brain areas involved in cognitive flexibility include but are not limited to the prefrontal cortex (PFC), motor association areas, posterior parietal cortex (PPC), anterior cingulate cortex (ACC), basal ganglia, and thalamus [15]. There are different types of sensory control that aids in switching pathways, like feedback and feedforward control. Comparative studies on adults and children showed that children had more switch-cost errors than adults, indicating the role of learning and memory in developing multiple pathways that are interconnected. The studies on the role of synaptic plasticity in cognitive and behavioral flexibility have shown that learning in an enriched environment can enhance cognitive flexibility.

## Discussion

The importance of multiple pathways in facilitating flexibility in movement and in the cognitive process can be helpful in understanding the principle underlying flexibility in decision-making. Human decision-making is also unpredictable and complex due to the large factors that can influence the vast network. It is important to recognize that no two brain networks can be identical when aiming to achieve the same goal. The flexibility arises from the diverse pathways involved in shaping individual behavior. Flexibility in neural networks has multiple advantages like providing variation among individual performances, adaptation, and possibility to improve the outcome. The flexibility in neural networks can also be traced to the stochastic differences in biomolecules and ion levels between cells. The complexity of neural pathways depends on the neurons involved, the synapses, the microenvironment of the neuron, the receptor-neurotransmitter interaction, levels of neurotransmitters, types of receptors, ionic transport, and the glial interaction. The molecular mechanism underlying memory formation is a good example to understand the neural mechanisms underlying behavior. Volition involves multiple options, and the presence of multiple pathways (both neural and cellular) might indicate the presence of volition or flexibility in decision-making.

The basic underlying principle of flexibility in neural networks and cellular signaling pathways involves the presence of multiple pathways with converging and diverging nodes. Thus, the authors would like to consider an alternative perspective on flexibility in decision-making as the result of multiple pathways that have convergence and divergence. Flexibility provides the possibility to have individualistic behavior, and the existence of multiple interconnected pathways in the cells can be considered as the molecular mechanism underlying individualistic decision-making in bacteria. The presence of such individualistic and collectivistic behavior, along with the ability for self-nonself discrimination, can indicate the presence of conscious decision-making in bacteria. The authors thus support the CBC because of the presence of conscious individualistic and collectivistic decision-making in bacteria. The author’s perspective on conscious decision-making in bacteria as a result of molecular events supports the ancient origin of consciousness and urges the importance of addressing the fundamental question of consciousness like ‘what constitutes the fundamental element/s of consciousness’.

## Data Availability

As new data was not generated or analyzed in this study, data sharing is not applicable.
